# Heather Nadean Holmes, AHIP, Medical Library Association President, 2025–2026

**DOI:** 10.5195/jmla.2026.2417

**Published:** 2026-07-01

**Authors:** Shannon D. Jones

**Affiliations:** 1 joneshan@musc.edu, Director of Libraries, Medical University of South Carolina, Charleston, SC

**Keywords:** Medical Library Association, MLA, Library Leadership, MLA President, Presidential Biography

## Abstract

This profile offers a portrait of Heather Nadean Holmes, MLIS, AHIP, 2025-2026 Medical Library Association (MLA) President, as a leader defined by authenticity, courage, and deep commitment to the profession. Holmes, known for her candor and dedication to the profession, brings a career rooted in service, gained from her work in hospital, academic, and association environments, to the presidency. Her professional journey reflects a sustained commitment to evidence-based practice, research, and elevating librarians as essential partners in health care, education, and discovery. Holmes is widely recognized for her work as a clinical medical librarian and her national leadership in developing the MLA Research Agenda. Colleagues describe her as a truth-teller who leads with integrity, sets clear boundaries, and consistently uses her platform to support others, especially early- and mid-career professionals. Her presidency focuses on inclusivity, transparency, and member engagement during a significant transition for both the Association and the profession. Beyond titles and accomplishments, this biography captures Holmes&s humanity. Her resilience, mentorship, humor, and unapologetic passions, including her love of K-pop, are highlighted. Together, these qualities portray a president who leads with conviction, empathy, and joy.

Whether she is curating a K-pop playlist or leading an organizational initiative, Heather N. Holmes is known for her energy, drive, and sass. As president, she brings those same qualities and a deep leadership experience to the Medical Library Association.

Heather was born and raised in Erie, Pennsylvania, where her early curiosity about the world took shape. As a child, she was deeply fascinated by ancient history, particularly ancient Greece and Egypt. She was captivated by the precision with which these civilizations built monumental structures and created enduring works of art without the benefit of modern tools, a level of ingenuity that still “makes my brain go into overdrive.” One of the earliest sparks for this interest came from quiet moments spent with her mother learning about the constellations and stars. It was an experience she now recognizes as foundational to her lifelong curiosity and sense of wonder.

As she imagined her future, librarianship was not yet on her radar. Instead, Heather’s aspirations shifted between becoming a teacher, an attorney, or a curator of antiquities. Above all, she dreamed of giving tours at museums and historical sites in Greece, sharing the stories of ancient places and the people who built them. She recalls feeling a profound sense of peace among ancient ruins and a deep enthusiasm for teaching others about their significance. That desire to help people learn, understand, and connect with information would later become a defining thread in her professional life.

**Figure:**
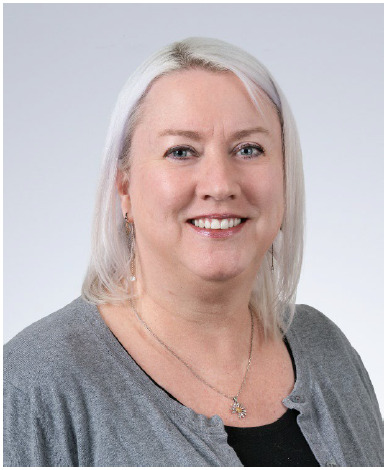


After graduating from college, Heather did not have a clear career path. She accepted a position as an evening library assistant at the Lake Erie College of Osteopathic Medicine (LECOM), where something clicked. “I did not fully understand or appreciate the level of knowledge or skills librarians had,” she recalls. However, as she spent time reshelving books and filing new acquisitions into the card catalog, she began to see the deeper power of organization, access, and discovery. “The lightbulb in my head went off in terms of discoverability of information and finding answers to things.” This moment of clarity led her to pursue her Master of Library and Information Science at the University of Pittsburgh. She later reflected that librarianship was not so different from one of her childhood dream jobs: being a tour guide in Greece. “It’s about leading people to something meaningful.”

Years later, she would find profound professional purpose when she began doing clinical librarianship full-time. “Many things came together for me then that made the work much more special,” she said. “I had the complete support of the entire leadership of the hospital, and I had complete confidence in what I was doing.” Gaining the respect of people who once saw her as “just a librarian” gave her the fuel to push further. “It made me want to be the best I could.”

Before joining the Medical University of South Carolina in 2016, where she now serves as Associate Director of Libraries and Professor, Heather held progressive roles in academic, law, and hospital libraries. She was best known professionally for her work as a hospital and clinical medical librarian. During her tenure at Summa Health System in Akron, Ohio, she developed an innovative clinical medical librarian program that took her out of the library and into daily bedside rounds, where she delivered real-time, evidence-based information in direct partnership with physicians. Her approach to embedded, point-of-care librarianship in a community-based academic medical center was highly unusual at the time and gained national recognition. In one case, her clinical searching helped diagnose arsenic poisoning in a patient who later made a full recovery. This work earned her the title “knowledge warrior”. It led to her being named a 2011 Library Journal Mover & Shaker in the Change Agent category for transforming how information professionals contribute to patient care [1]. In 2014, she received the Lois Ann Colaianni Award for Excellence and Achievement in Hospital Librarianship, MLA's highest honor for distinction in hospital library leadership, advocacy, and service.

A member of MLA since 2003, Heather has served on the Board of Directors from 2020 to 2023 and as Board Secretary from 2021 to 2023. She has chaired the Fellows and Honorary Members Jury, the Lindberg Research Fellowship Jury, and the Research and Evidence-Based Practice Curriculum Committee. She currently serves as Associate Editor for Hypothesis: Research Journal for Health Information Professionals. Heather’s presidency is grounded in a clear vision. She seeks to build an inclusive, collaborative, and member-focused association where diverse voices are heard, early and mid-career librarians are supported, and evidence and empathy guide decision-making. Her leadership style is marked by humility, clarity, and a deep commitment to elevating others.

A self-described fierce K-pop fan, Heather was drawn to the genre through friends who raved about “how amazing the dancing was and how the song lyrics were so impactful.” What began as curiosity quickly became passion:

“I was having a very hard time in both my work and professional life at that same time. It started by watching a couple videos, learning about the songs and their meanings, and that was all it took. I was completely drawn in.”

Her favorite group is ATEEZ, whom she has seen live eight times. Other favorites include NCT 127, SF9, Seventeen, and BTS. However, her ultimate bias is G-Dragon. As she puts it: “There is nobody on this earth that I love more than him (sorry, mom).”

K-pop has become more than a hobby. It is a source of emotional balance, creative energy, and personal grounding. Heather surrounds herself with K-pop photos and merchandise as “a constant reminder of things that make me happy.” She adds: “I am less reactionary than I used to be because of it.”

When asked how her fandom has shaped her work and leadership, she reflects:

“I always know that no matter what else is going on, I can listen to music or watch videos and escape the real world for a while. Their passion inspires mine.”

## GRIT, TRUTH, AND NAPKINS

From the moment I began working with Heather, her resilience struck me. She approaches challenges head-on, without pretense. What I admire most is her ability to be both honest and unflinchingly transparent. These qualities have earned her the trust of so many across our profession. Heather tells the truth, even when it is hard, with integrity, passion, and a deep love for librarianship. While she may come off as rough around the edges at times, that grit is matched by an unwavering commitment to the work and to the people doing it. Her leadership is real, grounded, and refreshingly human.

Since joining the MUSC faculty, Heather has also become a trusted confidante. She is someone with whom I have shared good laughs, reshaped library services, and exchanged countless ideas as a thought partner and lunch buddy. Most importantly, she is someone I call a friend. One of our favorite pastimes is deciding what we are doing for lunch, whether it is a quick walk to the hospital cafeteria, a visit to one of the food trucks on campus, or our go-to favorite, Swig & Swine. One thing you should know about Heather is that she loves napkins. Yes, napkins. Fancy ones, soft ones, hard ones, cheap ones. So, our lunches often turn into capers, as we search not only for good eats but also for the best disposable napkins. I always make sure to stuff my bag with extras for her enjoyment. Heather's collection of fountain pens is impressive, but it still does not hold a candle to her love for napkins.

You can tell a lot about a person based on how they love their animals. Heather is an animal lover through and through. A fond memory that I cherish to this day is the first time I met her fur son, Smoke. Smoke was a beautiful Sheltie with a gorgeous salt-and-pepper coat. At the time, I was not a dog person, but my timid petting of him that day unlocked something for me. About two weeks later, I became a pet parent myself to my own fur son, Cooper. They have both since crossed the rainbow bridge, but Smoke was something special. During the COVID-19 pandemic, one of my favorite moments was watching Smoke appear behind Heather during virtual meetings, quietly peering into the camera and patiently waiting to be let out into the backyard. It always made me chuckle, and I would ask, “What does Smoke want?” I miss seeing him standing there in the background. Today, Heather's fur family includes her beloved cats, Persephone and Jo, who continue to bring her comfort, companionship, and joy.

## IN THEIR VOICES: REFLECTIONS FROM COLLEAGUES.

Leadership is often best understood not only through titles or accomplishments, but through the people whose lives have been shaped by it. To know Heather, you must listen to the voices of those who have worked alongside her, learned from her, and witnessed her dedication firsthand. To help capture the essence of Heather’s leadership and impact, colleagues from across the profession were invited to contribute reflections based on the following prompt: *Can you contribute a short reflection on Heather’s leadership, impact, or her qualities as a colleague?*

What follows are their unedited responses, printed verbatim. Together, these reflections offer a portrait of a leader who is generous, strategic, grounded, and visionary. Through these voices, we come to know not just Heather Holmes, the president, but Heather Holmes, the colleague, thought-partner, mentor, and friend.

### Marie T. Ascher, MS, MPH, Lillian Hetrick Huber Endowed Director, Phillip Capozzi, M.D. Library, New York Medical College

Heather has been an integral part of establishing the Research Agenda since about 2011. At that time I introduced Heather to Jonathan Eldredge. I knew her just well enough to know she had a lot of drive as well as an interest in doing research — and we needed someone with a hospital background to join the Research Agenda Committee of the Research Section (now Caucus). This group was, at the time, working on the second iteration of the Research Agenda process. We've been working together on this project since, through an effort to facilitate groups in conducting systematic reviews related to the agenda through now when we encouraging research on the 15 questions emerging from the Agenda published in January 2025. What I've really come to value about working with Heather is her no-nonsense approach to large initiatives. She knows how to set boundaries and limits in order to get the work done and done well. She is also exceptional at bringing people together. She's one of those people who seems to know everyone and everything that is going on. She's a great person to bounce ideas off of and it often ends up with a new connection that begins with, “Do you know who you should talk to...?”

### Emily A. Brennan, MLIS, Evidence Synthesis Librarian, Professor, Academic Affairs Faculty, MUSC Libraries, Medical University of South Carolina

Heather is deeply committed to fairness, justice, and ensuring that individuals receive proper recognition and credit for their work. When I shared my frustration about being excluded as a co-author on a clinical guideline — despite my substantial contributions — due to an association rule limiting authorship to MDs, Heather took immediate and meaningful action.

She raised awareness of this inequity by engaging leadership across multiple organizations, including the MLA Board of Directors, MLA caucus chairs, and CHLA/ABSC. Her efforts led to the publication of the statement, “It’s Time to Acknowledge Authorship for Librarians and Information Professionals on Evidence Synthesis Publications.” This statement was subsequently shared with key authorship authorities, including the Committee on Publication Ethics (COPE) and the International Committee of Medical Journal Editors (ICMJE).

By issuing this statement, Heather not only elevated awareness of a long-standing issue, but also empowered librarians with a concrete tool to advocate for appropriate authorship recognition when engaging with editors and publishers. This experience exemplifies Heather’s leadership: she utilizes her position to lift others up, promote fairness and equity, and drive positive change for the profession.

### Jessica Diaz, MLIS, Research and Education Informationist, MUSC Libraries, Medical University of South Carolina

Heather is an exceptional leader. As a new medical librarian, her support and guidance have inspired me to pursue opportunities I wouldn't have been able to explore without her leadership. Whenever I have a new idea or revelation that excites me, I go into her office to share it because I know she will listen, consider my thoughts, and provide her insights—never dimming my light or enthusiasm. I feel incredibly fortunate to have Heather as my first supervisor in this profession. She has helped me develop a strong desire for challenges and instilled a fearlessness toward failure. With her support, I was able to launch the Southern Chapter’s Early Career Transitioning Librarians Initiative and share this mindset.

### Jonathan Eldredge, PhD, University of New Mexico

Heather joined the MLA Research Caucus’ Research Agenda Committee during 2011 when we were gearing-up for producing our next MLA Research Agenda. Heather was our sole hospital librarian on the Committee so we relied on her to represent her constituency, which she implemented faithfully. Heather had no research experience prior to joining so I was immediately impressed by her eagerness to learn everything about the research process. Her curiosity turned out to be insatiable. She must have asked me, alone, 100 questions about research. She even asked me about how I calculated probabilities! Later, Heather became famous for her “small research” advocacy and for her “Hypothesis Failure” column in *Hypothesis* that featured research projects that failed, but offered many lessons learned on what to avoid in one’s own future research projects”.

### Ryan Harris, MLIS, Associate Dean for Public Services, J. Murrey Atkins Library, UNC Charlotte

Heather has been an invaluable friend and colleague since I met her by a pool in Honolulu at the 2009 MLA Annual Meeting. Rough place to meet a new friend right? I was immediately comfortable with Heather from our first interaction and have never hesitated to reach out to her for advice. In particular as both of our career paths have led to higher and more complex levels of management and leadership, she has been an indispensable resource and colleague for me. She is someone I can reach out to when facing a challenging personnel issue or trying to navigate the constantly changing environment that is higher education. Sometimes work can give you unexpected roadblocks and you feel like you may not be doing a great job. I have often reached out to Heather to talk about these challenges. As her presidential address stated, you can learn from failure and challenges and she has helped me do so in several instances. Heather has never hesitated to let me reach out to her for guidance and support (and even to just vent a little). I know other health sciences librarians would say the same.

### Irene “Rena” Lubker, PhD, MLS, Research and Education Informationist, MUSC Libraries, Medical University of South Carolina

I don’t remember the exact year I first met Heather Holmes, but I do recall walking into my MUSC interview thinking, “If I nail this, Heather will be my supervisor,” and immediately trying to appear both highly qualified and like someone who can locate any citation. Heather became not just my supervisor, but also my friend who doesn’t make fun of my vertical challenges and is my unofficial therapist, listening with patience and dispensing wisdom that I actually take to heart. Some of that wisdom is straight from the Book of K-pop (“When in doubt, add glitter”), but it works, so who am I to argue?

I watched Heather work as the Associate Director of Libraries and a vital NNLM Region 2 Program Advisor without missing a beat. Later, she became the President of the Medical Library Association (MLA), navigating the change in leadership at MLA headquarters. Her CV reads like a box set: leader for MLA’s Research Agenda, Associate Editor for Hypothesis: Research Journal for Health Information Professionals, AAHSL Program & Education Committee contributor, MUSC Faculty Advisor to Presidential Scholars, and conductor of the RES department orchestra—seven informationists, one mission, and very few off-notes.

Beyond the titles, Heather is a very generous person, listens to me like a licensed counselor who moonlights as a librarian—calm, kind, and uncannily effective—then drops advice with the precision of a perfectly indexed database. She has also upgraded my pop-culture knowledge: I went from “Who’s BTS?” to “What is G-Dragon up to?” faster than you can say “bias.” She even hauled me into the cinematic universe of K-pop with a concert film starring Seventeen—yes, a group with thirteen members, Mingyu, Dino, and others. At concerts (and in movies about concerts), Heather proves that a person can mentor others, advance a profession, and still know exactly when to scream and have fun.

In short: Heather Holmes is living proof that you can run a library, guide a region, lead a national association, mentor your team, coach your anxious colleagues, convert them to K-pop, and still drop the mic—politely, on a soft surface, with proper citation.

### Teresa L. Knott, MLS, MPA, AHIP, FMLA *(she/her),* Associate Dean, VCU Libraries and Director, VCU Health Sciences Library, Virginia Commonwealth University

In the time I’ve known Heather Holmes, I have been struck by her passion for the profession. In particular, I’ve been impressed with her advocacy work. When she worked as a hospital librarian, she was a powerful voice for the importance of integrating clinical librarians into the care team. Then, Heather became a leading voice in the MLA Research Caucus. She advocated for implementing evidence-based librarianship and information practice. As part of this initiative, Heather strove to demystify a role for librarians conducting research by differentiating between “big R” research and “little r” research. To many MLA members, this framing changed their perspective on research into librarianship. In addition, she was an earlier contributor to the development of the MLA Research Training Institute, an initiative that continues to foster research into librarianship and building a generation of librarianship researchers.

Her leadership has been transforming MLA for many years. Throughout her career, Heather has been fearless and future focused. She strives to improve the status of librarians within organizations and to make the MLA a more member-focused organization. Heather is a person who will speak truth to power and always advocate for what she believes is the best way forward. She may also have an addiction to K-Pop, pets, and plants.

### J. Dale Prince, MA, MLS, AHIP, FMLA, Director, LSUHSC Libraries

I first met Heather Holmes at the 2009 MLA annual conference in Honolulu. We became close the following year, when we were both selected for the National Library of Medicine’s Biomedical Informatics Short Course in 2010. At that time, the course was offered twice a year at the Marine Biological Laboratory in Woods Hole, Massachusetts, during the laboratory’s off-season.

The program was an immersive experience: days filled with eight hours of lectures and hands-on work, evenings spent discussing the material at a pub after dinner, and nights sleeping in dormitory bunk beds. The cohort was intentionally interdisciplinary, bringing together librarians, physicians, scientists, and other healthcare professionals, with the expectation that participants would return to their institutions to share and apply what they had learned.

The curriculum covered major areas of biomedical informatics, including foundational concepts, electronic health records and clinical information systems, public health and consumer informatics, and, during the year we attended, disaster informatics, replacing the cellular biology systems usually covered. The work was intense, sometimes revelatory, and oriented toward innovation.

About halfway through the course, Heather remarked with genuine surprise, “I already do this.” To a degree, her reaction was understandable: informatics was, and often still is, framed as a highly technical specialty requiring formal, specialized training, which can give the impression that traditionally educated librarians are left on the margins, without a clear pathway to provide services that would meaningfully support their users. We’d both mistakenly had the idea that the Short Course was going to be an uphill battle from day one.

At the time, Heather was an Information Services Librarian at Summa Health System with twelve years of professional experience. Over that period, she had steadily honed her skills, staying current with emerging technologies and the evolving demands of the profession, and integrating complex concepts seamlessly into her daily work. In effect, she had already been practicing informatics without labeling it as such. In her characteristic modesty, she had underestimated her own capabilities and was genuinely surprised to recognize the breadth and depth of what she was able to do, having inadvertently trained herself in a demanding discipline while simply doing her job.

This response struck me as characteristic of Heather. She is not self-effacing or unaware of her strengths; upon returning home, she fully intended to discuss her expanded role with her employers and was, in fact, soon promoted. Rather, her modesty leads her to assume that the work she does is unexceptional and easily replicable, even when it clearly is not. That modesty masks a deep competence and a clear readiness for leadership, not a lack of confidence. Heather’s ability to quietly build expertise, recognize its broader value, and translate that insight into institutional advancement exemplifies a hallmark of effective leadership: practical innovation grounded in service.

### Tony Nguyen, MLIS, AHIP-D, Rutgers Health Libraries, Associate University Librarian, Rutgers University Libraries, Rutgers, The State University of New Jersey

One of the earliest opportunities I’ve had to engage with Heather Holmes was during the early start of my tenure working for the Network of the National Library of Medicine, back in 2015. I was keenly aware of the difficulties hospital librarians faced, particularly in becoming one-librarian service institutions or being closed due to budgetary cuts. I connected with Heather based on my knowledge of her commitment and passion for advocating on behalf of hospital librarians. The presentation she provided other hospital librarians at the time and opportunity to gain and share ideas on how they, too, could advocate for themselves within their hospital libraries. This early engagement with Heather proved to be a long-time frolleague who has often volunteered to share their insights and expertise while on various advisory committees over the years, showcasing her commitment to the profession and her desire to elevate the medical library profession.

Our collaborative engagement continued over the decade. During the pandemic, when the nation and the world shut down, Heather and I collaborated and co-chaired the first virtual Network of the National Library of Medicine symposium focused specifically on addressing COVID-19 and health misinformation. We both learned a great deal as we identified new ways to engage with the medical library profession. However, I believe that everyone who worked on the project ultimately left with a sense of pride and a feeling of accomplishment.

### MJ Tooey, Retired, Library Faculty Emerita, Health Sciences and Human Services Library, University of Maryland, Baltimore, Heather the Evangelist

When I first heard of Heather, I was told she was a bit rough around the edges. And when I finally met her as we worked on a committee imagining the future of the Medical Library Association, I found she was smart, engaged, outspoken, and fun to work with. Thus began our friendship.

Over the years I have come to regard as a person of passion, an evangelist. Whether it is for the medical library profession or her beloved K-pop entertainers, she is all in. She has been involved at all levels of our profession.

She has been a trusted hospital librarian, both by the profession and within the institutions she served. She was recognized as a “Mover and Shaker” in 2011 by the American Library Association. Truer words were never spoken and never was an award more deserved, appropriate, and prescient.

Moving into an academic setting in South Carolina, she moved out of her comfort zone and into the complex world of academic medicine. When the Medical University of South Carolina received the award to become Region 2 of the Network of the National Library of Medicine, she steadfastly served as its interim executive director, getting the program up and running and setting the standard for the executive directors following her.

Her passion for research led her to the editorship of Hypothesis, the publication of the Research Caucus. Throughout her career she has evangelized for quality research and for everyone to get involved whether in “big” research through grants and contracts, or “small” research impacting and improving daily library operations. Her evangelical adherence to the need for evidence-based practice is legendary and she is expert.

One cannot talk about Heather without commenting on her evangelical passion for K-pop. How would any of us know about Ateez or G-Dragon without her? They have appeared in many of her presentations and in many of her conversations. And she is not apologetic about her passion. She is a loyal friend. In my case, she married friendship and K-pop passion, with a trip to an Ateez concert in Baltimore. When I heard they were coming to Baltimore, I mentioned that maybe we could get together for dinner. However, Heather did me one better and invited me to attend the concert. Of course I did! When would I have another opportunity to enjoy the concert with a passionate, evangelical expert? It was amazing. She is an excellent friend.

Heather Holmes is also a person of her word. She is responsive. She does what she says she will do. As president of MLA, she has listened to the concerns of many whether it is about finances or communications or the importance of our profession. She approaches challenges logically, occasionally with colorful language, but always thoughtfully and respectfully. During her presidential year, there will be a new executive director, and she will see more transition with long-time, knowledgeable staff retirements. There are questions about professional associations. Sometimes people are in the right place at the right time and this is Heather’s time and place. Her advocacy, evangelism, and passion for medical libraries makes it so.

### Elizabeth C. Whipple, MLS, Interim Associate Dean and Director, Welch Medical Library, Johns Hopkins University & Medicine

Heather has been an advocate for inclusiveness within the profession. If you’ve known her for any amount of time, you most likely have heard her advocate that we should be the Health Sciences Library Association, reflecting the makeup of folks in our professional home, at schools of medicine or otherwise. Speaking of professional home, while this is not a new concept, Heather and I served MLA on the Futures Task Force together. I believe it was during a meeting for the MLA Futures Task Force when I was in a taxi (yes, not a Lyft or Uber) with Heather and the inestimable Max Anderson coined that phrase, “professional home”. We took that language back to the Futures Task Force, and I believe that through that synergy and conversation, other folks in MLA have now also adopted the language of MLA being our “professional home”.

Heather is also one of the foremost experts in our profession on using the delphi method in evidence based practice research for librarians. Her collaborative work resulted in the several articles on the new MLA Research Agenda, all published in JMLA, our premier journal for the profession. She began this work in 2008, the first paper was published in 2012, and most recently she published the 2024 Medical Library Association research agenda. Additionally, Heather has been recognized for her incredible work through being named as one of Library Journal’s Movers and Shakers in 2011, and her outstanding work as a hospital librarian through the Lois Ann Colaianni Award for Excellence and Achievement in Hospital Librarianship; both of these recognize her work making a difference in our profession.

While Heather and I are opposites in many ways—she is: introspective, a philosopher, a great gift giver; and I’m the opposite, she has always been there for big life changes for me, can make me laugh, and my professional and personal life would be lacking without her. She is an absolute credit to our profession and her advocacy is needed in this time of upheaval and uncertainty.

### JoAnn Zeise, MA, Curator, Waring Historical Library, MUSC Libraries, Medical University of South Carolina

Picture it: Charleston, South Carolina, 2023.

A new manager at a medical university library—someone who had never worked in a library or academia—sits quietly in a manager’s meeting at the Medical University of South Carolina’s Colbert Education Center and Library. A challenging topic is on the table, and conversations swirl. This newcomer isn’t going to speak up.

The group has no designated leader, yet one person steps forward—not with authority, but with experience, grace, and respect. They help guide the discussion, fostering a safe space through thoughtful dialogue. The new manager felt included and happy to be part of a team that supported each other and demonstrated the type of leadership they aspired to.

That new manager was me. The person who stepped forward was Heather Holmes.

Since that meeting, I have continued to learn from Heather’s example of leadership rooted in integrity, empathy, and vision. Her door has always been open—whether I needed advice, answers, or simply someone to listen. Even while she was temporarily managing a second team, Heather modeled grace and diligence, inspiring excellence throughout the library. She supported her team through uncertainty and change, encouraging them not just to cope, but to excel.

Heather’s influence extends far beyond her office. I’ve seen the passion that drives her volunteer work with local and national organizations. Juggling those roles alone deserves applause, not to mention the mentoring, team building, and initiatives she leads. As a board member for the Waring Library Society, where I work, I’ve witnessed her values in action. She champions the Waring team and our work, knowing when to send a supportive text, offer a listening ear, or be a sounding board. Through her actions, Heather demonstrates that authentic leadership means self-awareness, compassion, fostering learning, and inspiring others through integrity.

## CONCLUSION

Overall, there is collective admiration and appreciation for Heather’s leadership as MLA President. Reflecting on what she means to me, her colleagues, and our profession, I am reminded of an important truth: the best leaders do not just show up; they create opportunities for others to thrive as well. Heather has exemplified this through her advocacy and consideration for the Association's membership. She has encouraged us to laugh, grow, and embrace our authentic selves as information professionals. By infusing her vision and voice into key Association initiatives, she has demonstrated what it means to lead with integrity, connection, and care.

I am truly proud of her for how she has embraced the role of President. I am grateful that she has agreed to serve MLA in this capacity. The position of MLA President carries tremendous responsibility to both the membership and the profession. It requires resilience, tenacity, compassion, and decisive action. Most importantly, I want to thank her for leading in a way that demonstrates that professionalism does not necessarily equate to performance. It can also mean being authentic, present, and intentional. Impactful leaders do more than leave a mark; they light a path. I am committed to walking alongside her to ensure that Heather’s presidential year is energizing, inclusive, and impactful for both her and the association. I invite you to join me on this journey as we continue to show up together with purpose, joy, and courage.
